# In Vivo Treatment with a Standardized Green Tea Extract Restores Cardiomyocyte Contractility in Diabetic Rats by Improving Mitochondrial Function through SIRT1 Activation

**DOI:** 10.3390/ph15111337

**Published:** 2022-10-28

**Authors:** Rocchina Vilella, Simona Izzo, Valeria Naponelli, Monia Savi, Leonardo Bocchi, Cristina Dallabona, Maria Carla Gerra, Donatella Stilli, Saverio Bettuzzi

**Affiliations:** 1 Department of Chemistry, Life Sciences and Environmental Sustainability, University of Parma, 43124 Parma, Italy; 2 Department of Medicine and Surgery, University of Parma, 43125 Parma, Italy; 3 Adamas Biotech, 73024 Maglie, Italy; 4 National Institute of Biostructure and Biosystems (INBB), 00136 Rome, Italy

**Keywords:** diabetic cardiomyopathy, mitochondrial function, Sirtuin 1, calcium handling, green tea catechins, ATP, miRNAs

## Abstract

**Background.** Green tea catechins are known to promote mitochondrial function, and to modulate gene expression and signalling pathways that are altered in the diabetic heart. We thus evaluated the effectiveness of the in vivo administration of a standardized green tea extract (GTE) in restoring cardiac performance, in a rat model of early streptozotocin-induced diabetes, with a focus on the underlying mechanisms. **Methods.** Twenty-five male adult Wistar rats were studied: the control (n = 9), untreated diabetic animals (n = 7) and diabetic rats subjected to daily GTE administration for 28 days (n = 9). Isolated ventricular cardiomyocytes were used for ex vivo measurements of cell mechanics and calcium transients, and molecular assays, including the analysis of functional protein and specific miRNA expression. **Results.** GTE treatment induced an almost complete recovery of cardiomyocyte contractility that was markedly impaired in the diabetic cells, by preserving mitochondrial function and energy availability, and modulating the expression of the sarcoplasmic reticulum calcium ATPase and phospholamban. Increased Sirtuin 1 (SIRT1) expression and activity substantially contributed to the observed cardioprotective effects. **Conclusions.** The data supported the hypothesis that green tea dietary polyphenols, by targeting SIRT1, can constitute an adjuvant strategy for counteracting the initial damage of the diabetic heart, before the occurrence of diabetic cardiomyopathy.

## 1. Introduction

Diabetic cardiomyopathy (DCM) is a multifactorial specific heart disease that develops in more than 50% of both type 1 and type 2 diabetic subjects, independently of the coexistence of other cardiac pathologies [1]. Multiple mechanisms contribute to the pathogenesis of DCM, including cell oxidative stress, moderate myocardial tissue inflammation, mitochondrial dysfunction, apoptotic cell loss and altered signalling pathways, progressively leading to abnormalities in cardiomyocyte contractile properties and calcium homeostasis, and eventually to ventricular dysfunction [1,2,3,4]. A growing number of studies have also focused on the key role of epigenetic factors in the aetiology of DCM. Among these, major attention has been devoted to the diabetes-induced decreased expression of the NAD+-dependent histone deacetylase, Sirtuin 1 (SIRT1) [5], as well as to changes in the physiological levels of specific, non-coding, single-stranded microRNAs [6]. SIRT1, besides histones, can deacetylate several transcription factors to regulate the expression of target genes and can also act on cytosolic proteins, among which is the sarcoendoplasmic reticulum calcium ATPase (SERCA2), the most important player involved in cardiomyocyte calcium dynamics [7]. More recently, the upregulation or downregulation of a number of miRNAs has been described in the diabetic heart of both experimental models and human subjects [1,5,8]. These changes lead to the posttranscriptional dysregulation of target genes that modulate mitochondrial function, oxidative stress response, inflammatory processes, cell survival and excitation–contraction processes.

Despite the significant research attention devoted through several decades to the pathogenic mechanisms underlying multifactorial, diabetes-induced cardiomyopathy, effective treatments able to prevent the initial changes occurring in the diabetic heart, before the appearance of overt signs of cardiac dysfunction, are still not available [1].

The promising results previously obtained in experimental models of early diabetes suggest that natural polyphenolic compounds could constitute an adjuvant therapeutic tool. Many of these compounds are able to restore the intracellular signalling pathways, which are altered at the initial stages of diabetes, leading to a recovery of cardiac functional properties [9,10,11,12]. So far, less documented are the effects of the polyphenolic compounds in counteracting the changes in the expression of specific miRNAs, and their target genes, involved in the pathogenesis of DCM.

Among the dietary polyphenols, green tea catechins have been reported to produce metabolically beneficial effects by promoting the expression and activity of SIRT1 and 5′ AMP-activated protein kinase (AMPK) [13]. SIRT1 and AMPK, whose levels are decreased in the diabetic heart, regulate each other and share many common target molecules, such as the transcriptional coactivator, peroxisome proliferator-activated receptor gamma coactivator 1-alpha (PGC1-alpha), which is the main regulator of mitochondrial biogenesis and function [13]. The two enzymes also modulate several transcription factors leading to the activation of antioxidant response elements (ARE) and the inhibition of the NF-kB-mediated proinflammatory pathway [14]. We recently showed that a standardized green tea extract (GTE; Theaphenon-E) [15], given in vivo to healthy rats, enhanced cardiomyocyte contractile efficiency by increasing mitochondrial function and energy availability, through the enhancement of the level of the OXPHOS complexes, and by targeting key excitation–contraction proteins [16].

Based on the above considerations, in the present study, we evaluated the effectiveness of GTE oral administration in restoring cardiac performance in a rat model of early streptozotocin-induced diabetes, with a focus on the underlying mechanisms. Specifically, we tested whether GTE was capable of restoring cardiomyocyte ATP content, mitochondrial metabolic activity and SIRT1 levels. Further, we analysed whether specific microRNAs, potentially linked to the initial alterations of the diabetic myocardium, are already dysregulated at the initial stages of diabetes [17,18]. Specific attention was devoted to miRNA1, miRNA22 and miRNA34a, which were shown to be involved in the modulation of the expression of SIRT1 [19,20], as well as of genes related to intracellular calcium handling [21,22,23]. The functional counterpart of these actions was determined at the cellular level by measuring cardiomyocyte contractile properties and calcium dynamics.

## 2. Materials and Methods

The investigation was approved by the Veterinary Animal Care and Use Committee of the University of Parma in Italy and met the National Ethical Guidelines of the Italian Ministry of Health (Prot. N°614/2016-PR).

### 2.1. Animals and Experimental Protocol

The study was performed on 25 male Wistar rats (Rattus norvegicus) aged 14–16 weeks, weighing 414 ± 9.6 g (mean ± standard error of the mean), individually housed in a temperature-controlled room at 22–24 °C, with the light on between 7 a.m. and 7 p.m. The bedding of the cages consisted of wood shavings, and food was freely available (Mucedola S.r.l., Milan, Italy). Nine rats were used as the control group (CTRL) while type-1 diabetes was induced in the remaining sixteen animals (group D) by a single intra-peritoneal (i.p.) injection of streptozotocin (STZ, 60 mg/kg; Sigma-Aldrich, Milan, Italy) [11]. Glucose blood levels and body weights were measured in 4h-fasting animals before and two days after the STZ injection, and then weekly until sacrifice. After the documented increase in blood glucose levels (2 days after STZ injection; blood glucose cut-off: 250 mg/dL), D animals were either untreated (D group, n = 7) or subjected to daily green tea extract (GTE; Theaphenon-E) oral administration (90 mg/day of GTE dissolved in 50 mL of tap water) for 28 days (D_GTE, n = 9) [16]. The chemistry, purification, development and validation of the GTE used in our study have already been described [15]. Briefly, catechins are purified from green tea leaves by extraction with water first and then with ethyl acetate, followed by column chromatography using water/alcohol to elute the catechins. The resulting catechin powder, called Polyphenon E (or Theaphenon E), with a total catechin content of about 90%, is composed of epigallocatechin gallate (EGCG, 68.58%), epigallocatechin (EGC, 10.56%), epicatechin gallate (ECG, 5.95%), epicatechin (EC, 4.31 %), and traces of caffeine, theobromine and gallic acid. Polyphenon E is a trademark of Mitsui Norin Co., Ltd., Tokyo, Japan and Theaphenon E is a trademark of Tea Solutions, Hara Office Inc., Tokyo, Japan.

At the end of the experimental protocol, the animals were anesthetized with ketamine chloride (Imalgene; Merial, Milan, Italy; 40 mg/kg i.p.) plus medetomidine hydrochloride (Domitor; Pfizer Italia S.r.l., Latina, Italy; 0.15 mg/kg i.p.), and sacrificed by decapitation. In 14 animals (five CTRL, four D and five D_GTE), the heart was rapidly excised, and ventricular cardiomyocytes were enzymatically isolated for the ex vivo measurement of cell mechanical properties and calcium transients. A fraction of isolated left ventricular myocytes was washed three times with low-calcium solution (0.1 mM) and centrifuged (42× *g* for 5 min). After removing the supernatant, the pellet was stored at −80 °C. From the remaining rats (four CTRL, three D and four D_GTE), the heart was excised and perfused with a 0.9% NaCl solution at 37 °C to drain the residual blood. Then, the tissues (left and right ventricles) were snap frozen in liquid nitrogen and stored at −80 °C. The stored cardiomyocytes and tissue samples were used for biochemical and molecular analyses.

### 2.2. Isolation of Adult Left Ventricular Cardiomyocytes

Individual left ventricular cardiomyocytes were enzymatically isolated by collagenase perfusion, in accordance with a procedure previously described [11,16]. Briefly, the heart was mounted on a Langendorff apparatus and retrogradely perfused at 37 °C through the aorta with the following sequence of solutions gassed with 100% oxygen: (1) a calcium-free solution for 5 min to remove the blood, (2) a low-calcium solution (0.1 mM) plus 1 mg/mL of type 2 collagenase (Worthington Biochemical Corporation, Lakewood, NJ, USA) and 0.1 mg/mL of type XIV protease (Sigma-Aldrich) for about 15 min, and (3) an enzyme-free, low-calcium solution for 5 min. The calcium-free solution contained the following (in mM, all chemicals from Sigma-Aldrich): 126 NaCl, 22 dextrose, 5.0 MgCl_2_, 4.4 KCl, 20 taurine, 5 creatine, 5 Na pyruvate, 1 NaH_2_PO_4_ and 24 HEPES (pH = 7.4, adjusted with NaOH). The left ventricle was then minced and shaken for 10 min. The cells were filtered through a nylon mesh and a fraction was resuspended in low-calcium solutions for 20 min, gradually brought to 1mM of Ca^2+^ in about 80 min, and then used for measuring sarcomere shortening and calcium transients.

### 2.3. Cardiomyocyte Contractility and Calcium Transients

Mechanical properties and calcium transients were evaluated by using the IonOptix fluorescence and contractility systems (IonOptix, Milton, MA, USA). Left ventricular myocytes were placed in a chamber mounted on the stage of an inverted microscope (Nikon-Eclipse TE2000-U; Nikon Instruments, Florence, Italy) and superfused (1 mL/min at 37 °C) with a Tyrode solution containing (in mM, all chemicals from Sigma-Aldrich): 140 NaCl, 5.4 KCl, 1 MgCl_2_, 5 HEPES, 5.5 glucose and 1 CaCl_2_ (pH 7.4, adjusted with NaOH). Only rod-shaped myocytes with clear edges and an average sarcomere length ≥ 1.7 μm were selected for the analysis, using a 40× oil objective lens (NA:1.3). None of the selected myocytes showed spontaneous contractions. The cells were field stimulated at a frequency of 0.5 Hz by constant current pulses (2 ms in duration and twice the diastolic threshold in intensity; MyoPacer Field Stimulator; IonOptix), delivered by platinum electrodes placed on opposite sides of the chamber, connected to a MyoPacer Field Stimulator (IonOptix). Load-free contractions of myocytes were measured with the IonOptix system, which captures sarcomere length dynamics via a fast Fourier transform algorithm [16].

Cell contractile properties and calcium dynamics were simultaneously recorded, after loading the myocytes with fluo-3 AM (5 μM; Thermo Fisher Scientific, Waltham, MA, USA), previously mixed with PluronicTM F-127 (10% final concentration; Thermo Fisher Scientific, Waltham, MA, USA), for 20 min.

In a total of 170 left ventricular myocytes (59 CTRL, 56 D and 55 D_GTE), the following parameters were assessed: mean diastolic sarcomere length (BL); the fraction of shortening (FS); maximal rates of shortening (−dL/dt_max_) and relengthening (+dL/dt_max_); time to 90% of relengthening (RL90%); the amplitude of the calcium transient, expressed as normalized fluorescence (f/f0); and the time constant (τ) of the fluorescence signal decay, as an index of the rate of intracellular calcium clearing [24].

### 2.4. ATP Content Analysis in Isolated Cardiomyocytes

The ATP intracellular content was measured by the CellTiter-Glo Luminescent Cell Viability Assay (Promega, Milan, Italy) according to the manufacturer’s protocol. Briefly, a frozen pellet of isolated cardiomyocytes was resuspended in 1 mL of phosphate-buffered saline (PBS); 5 µL of this suspension was further diluted to a final volume of 400 µL with PBS. An amount of 100 µL of the diluted cell suspension was aliquoted in triplicate in a 96-well white plate and lysed with 100 µL of mammalian cell lysis solution for 2 min in an orbital shaker. The microplate was incubated for 10 min at room temperature before measuring the luminescence intensity by the EnSpire Multimode Plate Reader (PerkinElmer, Inc., Waltham, MA, USA). The row luminescence data, given in relative light units (RLUs), were normalized to the total protein content of each sample, measured by the DC Protein Assay Kit (Bio-Rad, Hercules, CA, USA).

### 2.5. Citrate Synthase Activity Assay

The citrate synthase activity of the rat heart tissue was detected by using MitoCheck Citrate Synthase Activity Assay Kit (Cayman Chemical, Ann Arbor, MI, USA), according to the manufacturer’s protocol. An aliquot of nitrogen-ground rat heart tissue was resuspended in 100 µL of ice-cold radioimmunoprecipitation assay buffer (RIPA), supplemented with adequate amounts of protease and phosphatase inhibitor cocktails (Sigma-Aldrich, Milan, Italy). An aliquot of 100 μg/mL of CTRL, D and D_GTE tissue extract prepared in kit assay buffer was diluted 50 times, and the absorbance intensity was measured at 30 s intervals for 20 min at 25 °C using the EnSpire Multimode Plate Reader (PerkinElmer, Inc.). Citrate synthase catalyses the condensation of the dicarboxylate oxaloacetate and acetyl-CoA to the tricarboxylate citrate. The assay measures the production of SH-CoA by monitoring the absorbance of Citrate Synthase Developing Reagent at 412 nm. One unit of citrate synthase turns over 1 mmol of developer per minute at 25 °C, pH 7.4.

### 2.6. SIRT1 Deacetylase Activity Assay

SIRT1 activity was determined in extracts from the heart tissues by using an SIRT1 fluorometric assay kit (Abcam, Cambridge, UK), according to the manufacturer’s instructions. The fluorescence intensity (Ex355 nm/Em460 nm) was measured at 2-min intervals for 60 min using the EnSpire Multimode Plate Reader (PerkinElmer, Inc.). Briefly, a few milligrams of liquid nitrogen-ground CTRL, D and D_GTE rat left ventricles were homogenized in 100 µL of ice-cold radioimmunoprecipitation assay buffer (RIPA). The protein lysate was diluted to a final concentration of 500 µg/mL in kit assay buffer and 15 µL was used for the assay. The reaction was followed for 60 min and the values of fluorescence vs. the time of reaction were plotted. The activity was calculated through the slope of the curve.

### 2.7. RNA Extraction, cDNA Synthesis and Relative Expression of Genes

CTRL, D and D_GTE rat ventricles were ground in liquid nitrogen to a fine powder. Ten mg of tissue powder was lysed with 1 mL of TRIzol reagent (Thermo Fisher Scientific, Waltham, MA, USA), purified with the PureLink RNA Mini Kit (Thermo Fisher Scientific), and reverse transcribed using the RevertAid First Strand cDNA Synthesis Kit (Thermo Fisher Scientific), according to the manufacturer’s instructions. Briefly, 500 ng of total purified RNA was added with 1 µL of Random Primers (100 µM) and heated at 65 °C for 5 min. The first-strand synthesis reaction was carried out through the incubation of each ice-chilled sample for 60 min at 42 °C and stopped at 70 °C for 5 min. The obtained cDNA was used for qPCR amplification by the SsoAdvanced Universal SYBR Green Supermix (Bio-Rad), using the specific primers reported in Table 1. Each cDNA sample was run in duplicate on a DNA Engine Opticon 4 (MJ Research, Waltham, MA, USA). Thermal cycler conditions consisted of an initial denaturation at 95 °C for 30 s, followed by 40 cycles of denaturation at 95 °C for 15 s, and annealing and extension at 60 °C for 20 s. The cycle threshold (Ct) for each target gene was normalized to the Ct value of the reference gene, glyceraldehyde phosphate dehydrogenase (GAPDH), according to the following equation: normalized Ct (∆Ct) = Ct target gene − Ct GAPDH [25,26].

### 2.8. Protein Extraction, SDS–Polyacrylamide Gel Electrophoresis (SDS–PAGE) and Western Blot (WB) Analysis

Thirty mg of liquid nitrogen-grounded CTRL, D and D_GTE rat ventricles was used to extract proteins in 500 µL of ice-cold modified RIPA buffer (50 mM Tris HCl pH 7.4, 100 mM NaCl, 1% Triton X-100), supplemented with an adequate amount of protease and phosphatase inhibitor cocktails (Sigma-Aldrich). The supernatants of the extracts obtained by centrifugation (14,000× *g* rpm for 30 min at 4 °C) were stored at −20 °C. Protein concentration was estimated by the DC Protein Assay Kit (Bio-Rad), using bovine serum albumin (Sigma-Aldrich) as a standard. The protein lysate (10–30 µg) was separated by SDS–PAGE and blotted onto PVDF membranes (EMD Millipore, Merk KGaA, Darmstadt, Germany). Transfer efficiency was routinely monitored by 0.1% Ponceau S staining (Sigma-Aldrich). After blocking in a solution of 5% milk, the blotted membranes were incubated with primary antibodies overnight at 4 °C: rabbit polyclonal anti-phospho-phospholamban (Ser16) (EMD Millipore Corporation, Temecula, CA, USA, code 07-052), dilution 1:200; rabbit polyclonal anti-phospholamban (Abcam, Cambridge, UK, code 126174), dilution 1:500; rabbit polyclonal anti-SERCA2 (Abcam, Cambridge, UK, code ab3625), dilution 1:2000; rabbit monoclonal anti-SIRT1 (Abcam, Cambridge, UK, code 189494), dilution 1:3000; and mouse monoclonal anti-actin (Santa-Cruz Biotechnology, Santa Cruz, CA, USA), dilution 1:500. Immunoreactive bands were detected using BM Chemiluminescence Western Blotting Substrate (Hoffmann-La Roche, Basel, Switzerland) and quantified by Quantity Basic analysis Software (Bio-Rad).

### 2.9. RNA Extraction and cDNA Synthesis

Disruption and homogenization of the left ventricular tissues was performed using an ultrasonic probe sonicator. Total RNAs were extracted using the miRNeasy Tissue/Cells Advanced Mini Kit (Qiagen, Hilden, Germany), according to the manufacturer’s instructions, and DNase1 (RNase-Free DNase Set; Qiagen) was used to eliminate DNA contamination for 45 min at 37 °C. The quantity of total RNA extracted was determined using a NanoDrop 1000 Spectrophotometer (Thermo Fisher Scientific, Waltham, MA, USA).

cDNA was synthesized using the TaqMan MicroRNA Reverse Transcription Kit. Each reaction consisted of 5 μL of total RNA and 7 μL of the reverse transcription reaction mix, including 0.15 μL of 100 mM dNTPs, 1 μL of 50 U/μL of MultiScribe Reverse Transcriptase, 1.5 μL of reverse transcription buffer, 0.19 μL of 20 U/μL of RNase Inhibitor (Applied Biosystems, Waltham, MA, USA; Thermo Fisher Scientific) and 4.16 μL of nuclease-free water. A total of 3 μL of 5X TaqMan miRNA-specific stem-loop primers (Applied Biosystems; Thermo Fisher Scientific) was added to each reaction tube, for a total volume reaction of 15 μL. The reverse transcription reaction mixture was incubated at 16 °C for 30 min, at 42 °C for 30 min and at 85 °C for 5 min, and then was held at 4 °C. Negative controls were also included in the experiment to exclude genomic DNA contamination.

### 2.10. MiRNA Expression Analysis

The relative expression of miRNAs was measured in triplicate using the real-time polymerase chain reaction (real-time PCR) technique, performed on an automatic QuantStudio 3 Real-Time PCR thermocycler instrument (Applied Biosystems), using TaqMan Fast Advanced Master Mix and TaqMan probes (20×) (Assay name: rno-miR22-3p, Assay ID: 000398; rno-miR34a-5p, Assay ID 000426; rno-miR1-3p, Assay ID 002064; Thermo Fisher Scientific, Waltham, MA, USA), according to the manufacturer’s instructions. The total reaction volume was 20 μL per well, in a 96-well optical plate. The expression levels of miRNA1, miRNA22 and miRNA34a were analysed using the following reaction conditions: enzyme activation at 95 °C for 20 s, followed by 40 cycles for denaturation at 95 °C for 1 s and annealing/extension at 60 °C for 20 s. U6 snRNA (Assay ID 001973; Thermo Fisher Scientific, Waltham, MA, USA) served as a housekeeping gene, i.e., the endogenous control [27,28]. Relative miRNA expression levels of the target genes were calculated as the fold change in each group (CTRL, D and D_GTE), using the delta-delta-Ct method (2^−ΔΔCt^) [29]. All reactions were run in triplicate and Ct data were determined using a cut-off value at Ct = 40, with U6 as an endogenous control.

### 2.11. Statistical Analysis

The IBM SPSS statistical package (version 28; International Business Machines Corporation, Armonk, NY, USA) was used. Normal distribution of variables was checked by means of the Kolmogorov–Smirnov test. Data were reported as mean values ± standard error of the mean (SEM), or as median values with interquartile ranges, as indicated in the figure captions. Comparisons among groups involved GLM ANOVA for repeated measurements (cell mechanics, calcium transients and intracellular ATP levels), followed by a Šidák post hoc test. Statistics of all molecular data included two-way ANOVA (Šidák post hoc test) or the non-parametric Kruskal–Wallis test (followed by the Mann–Whitney U test) when appropriate, as specifically reported in the figure legends. Differences were considered statistically significant at *p* < 0.05.

## 3. Results

### 3.1. Blood Glucose Levels and Body Weight

Two days after STZ injection, blood glucose levels significantly increased in diabetic rats compared with the CTRL (356 ± 11 mg/dL vs. 105 ± 4 mg/dL, respectively; *p* < 0.01). During the subsequent weeks, glycaemia increased slightly in untreated as well as GTE-treated diabetic animals, while it remained stable in the CTRL group until the end of the experimental protocol (Table 2). An approximately 10% decrease in body weights occurred during the first two weeks after STZ injection in both the treated and untreated diabetic rats. Subsequently, body mass exhibited only negligible changes while in CTRL animals, it slightly increased (Table 3).

### 3.2. Cardiomyocyte Mechanics and Calcium Transients

**Figure 1:**
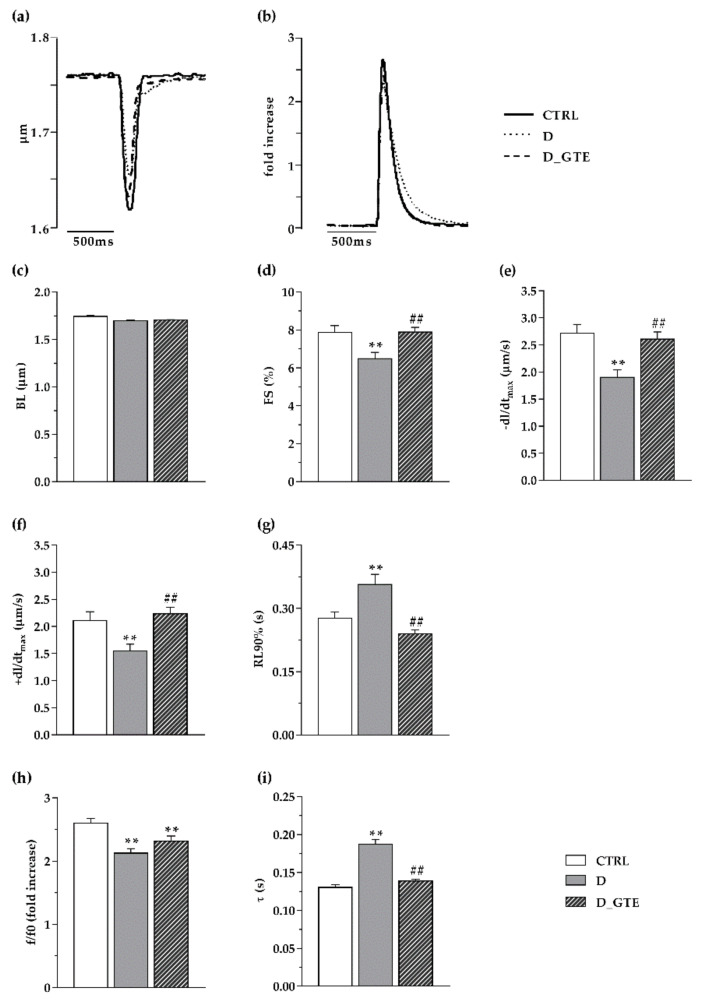
Cell mechanics and calcium transients. Representative examples of (**a**) sarcomere shortening and (**b**) corresponding calcium transients (normalized traces: fold increase) recorded from CTRL (solid line), D (dotted line) and D_GTE (dashed line) ventricular myocytes. In the c–i bar graphs, the mean values ± SEM of (**c**) mean diastolic sarcomere length (BL), (**d**) sarcomere fraction of shortening (FS), (**e**) maximal rate of shortening (−dL/dt_max_), (**f**) maximal rate of relengthening (+dL/dt_max_), (**g**) time to 90% of relengthening (RL90%), (**h**) calcium transient amplitude, expressed as peak fluorescence, normalized to baseline fluorescence (f/f0), and (**i**) time constant of the intracellular calcium decay (τ), measured in CTRL (59 cells), D (56 cells) and D_GTE (55 cells). ** *p* < 0.01 significant differences vs. CTRL; ## *p* < 0.01 significant differences vs. D (GLM ANOVA for repeated measurements).

In the absence of marked changes in the average diastolic sarcomere length (Figure 1c), contraction/relaxation properties and intracellular calcium transients worsened in unloaded ventricular myocytes isolated from D hearts in comparison with CTRL (Figure 1a–i). Specifically, D cardiomyocytes exhibited a significant decrease in the sarcomere fraction of shortening (FS, −17%; Figure 1d), the maximal rate of shortening (−dL/dt_max_, −27%; Figure 1e) and relengthening (+dL/dt_max_, −31%; Figure 1f), and the time to 90% of relengthening (RL90%, −13%; Figure 1g). The impaired contractility in D cells was accompanied by a significant decrease in the calcium transient amplitude (f/f0, −18%; Figure 1h) associated with higher values of the time required for cytosolic calcium clearing (τ, +44%; Figure 1i). In vivo GTE treatment led to an almost complete recovery of cellular mechanical properties and calcium dynamics which attained values comparable to those measured in the CTRL group (Figure 1a–i).

### 3.3. ATP Content Analysis

**Figure 2:**
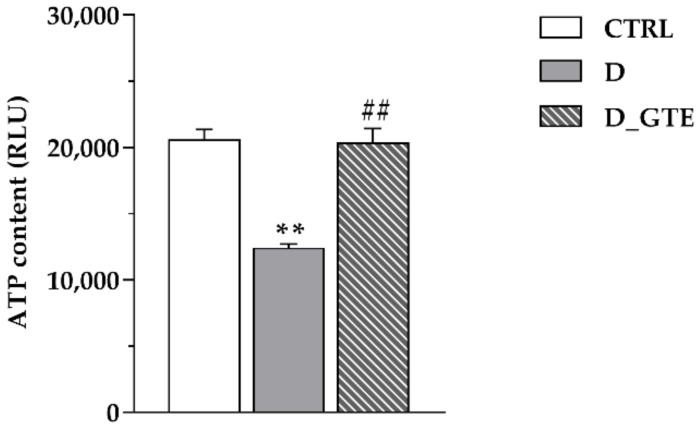
Mean values ± SEM of ATP content measured in CTRL, D and D_GTE cardiomyocytes. Values are expressed as relative light units (RLUs), normalized to the protein content. ** *p* < 0.01 significant differences vs. CTRL; ## *p* < 0.01 significant differences vs. D (GLM ANOVA for repeated measurements).

As compared with the controls, the ATP concentration in cardiomyocytes isolated from untreated diabetic rat hearts was significantly reduced (−40%, *p* < 0.001; Figure 2), but completely restored in D_GTE cells.

### 3.4. Citrate Synthase Activity

**Figure 3:**
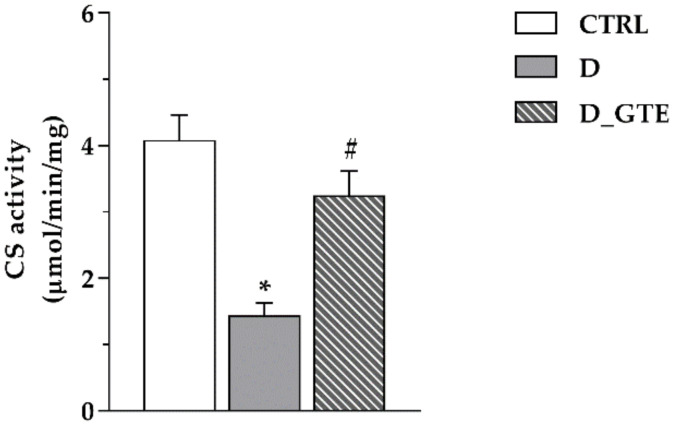
Citrate synthase activity of CTRL, D and D_GTE reported as µmol/min/mg. Histograms show the mean ± SEM. * *p* < 0.05 significant differences vs. CTRL; # *p* < 0.05 significant differences vs. D (Kruskal–Wallis test).

To test whether the increase in the ATP levels was associated with increases in mitochondrial mass, the enzymatic activity of citrate synthase (CS), which is a marker of mitochondrial mass [30], was measured in the heart homogenates of four CTRL, three D and four D_GTE left ventricular samples. Compared with control tissues, CS activity exhibited a three-fold decrease in D samples and an almost complete recovery in tissue samples of GTE-treated animals (Figure 3).

### 3.5. RT-qPCR Analysis of SERCA2, PLB, CACNA1C, NCX1 and RYR2 

**Figure 4:**
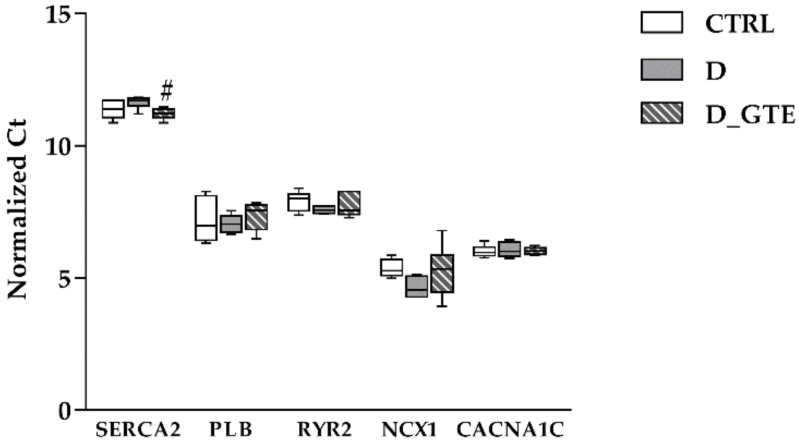
Box plot distribution of the normalized CTRL, D and D_GTE Ct values of SERCA2, PLB, RYR2, NCX1 and CACNA1C. The Ct value of the reference gene was normalized over the Ct value of GAPDH. Normalized Ct values: higher values correspond to lower gene expression levels; # *p* < 0.05 significant differences vs. D (Kruskal–Wallis test).

To verify whether GTE exerted a transcriptional modulation of the expression of proteins involved in the intracellular calcium handling, we analysed the steady-state levels of the mRNA of SERCA2, phospholamban (PLB), plasmalemma voltage-gated calcium channel (CACNA1C), sodium–calcium exchanger (NCX1) and ryanodine receptor 2 (RYR2). SERCA2 mRNA levels exhibited a slight decrease in the D animals as compared with the CTRL (Figure 4), while they were significantly increased in D_GTE (*p* < 0.05 vs. D). Conversely, no statistically significant changes among the groups were observed in the expression of PLB, RYR2, NCX1 or CACNA1C.

### 3.6. Western Blot Analysis

**Figure 5:**
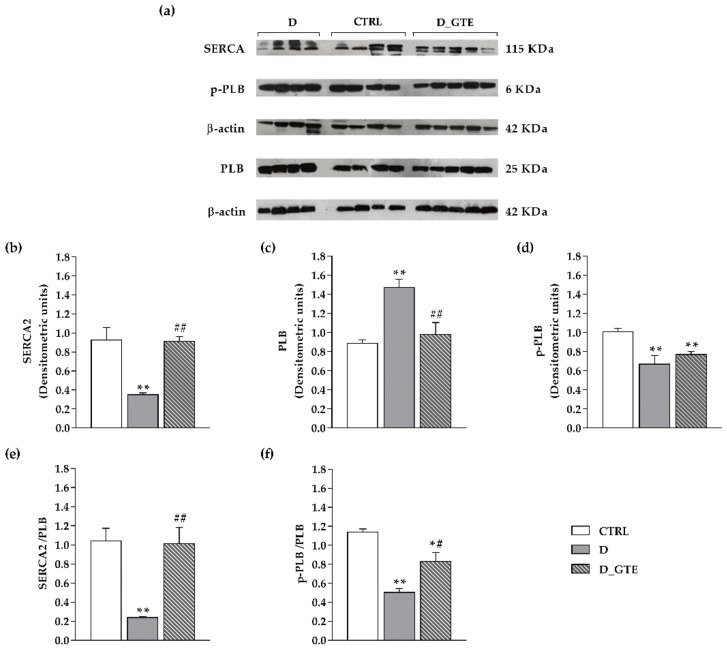
SERCA2, PLB, and p-PLB protein expression levels. (**a**) Typical electrophoretic separation and immunodetection of SERCA2, PLB and p-PLB in the heart tissue of CTRL, D and D_GTE rats. The expression levels (densitometric units) of (**b**) SERCA2, (**c**) PLB and (**d**) p-PLB were measured by densitometric analysis and normalized to b-actin. (**e**) SERCA2/PLB and (**f**) p-PLB/PLB. Histograms show the mean ± SEM. ** *p* < 0.01 significant differences vs. CTRL; * *p* < 0.05 significant differences vs. CTRL; ## *p* < 0.01 significant differences vs. D; # *p* < 0.05 significant differences vs. D (two-way ANOVA).

In order to evaluate the protein level of SERCA2, PLB and the phosphorylated form of PLB (p-PLB), we performed WB analyses (Figure 5). Compared with CTRL, D cardiomyocytes exhibited a significant decrease in SERCA2 protein level (−60%), a marked increase in total PLB (+66%) and a decrease in p-PLB (−33.6%), leading to a significant reduction in both the SERCA2/PLB and p-PLB/PLB ratios. All together, these changes can be considered responsible for the reduced contractile efficiency of D cells [31]. In vivo GTE administration restored the protein levels, with the exception of p-PLB, leading to a recovery of the values of the two ratios (SERCA2/PLB and p-PLB/PLB) which reached values very close to those measured in the CTRL cardiomyocytes.

### 3.7. SIRT1 Activity, Protein and mRNA Levels

**Figure 6:**
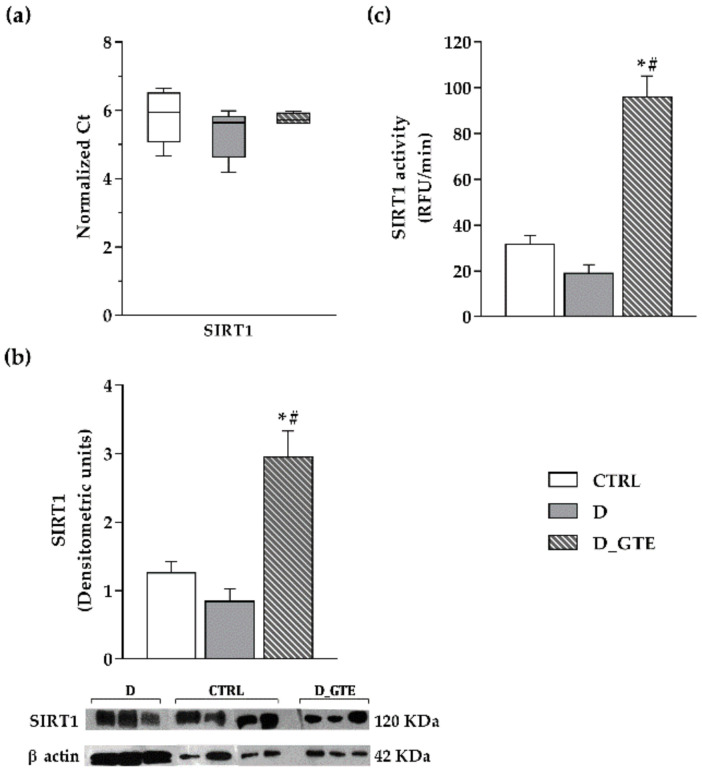
SIRT1 mRNA and protein expression levels and activity. (**a**) Box plot distribution of normalized CTRL, D and D_GTE Ct values of SIRT1. The Ct value of the reference gene was normalized over the Ct value of GAPDH. Higher normalized Ct values correspond to lower gene expression levels; (**b**) Typical electrophoretic separation and immunodetection of SIRT1 in the heart tissue of CTRL, D and D_GTE rats. The expression levels (densitometric units) of SIRT1 were measured by densitometric analysis and normalized to β-actin. (**c**) SIRT1 activity determined in CTRL, D and D_GTE rat heart tissue. The activity was reported as relative fluorescence units (RFU)/min. Histograms show the mean ± SEM. * *p* < 0.05 significant differences vs. CTRL; # *p* < 0.05 significant differences vs. D (Kruskal–Wallis test).

SIRT1 expression, both at the mRNA and protein levels, and SIRT1 activity did not show any significant differences between the D and CTRL groups, with only slight changes in D (Figure 6a,b). Conversely, in D_GTE left ventricular samples, a significant increase was observed in SIRT1 protein level and activity (Figure 6b,c), as compared with both the CTRL and D groups, suggesting a posttranscriptional modulation of SIRT1.

### 3.8. MicroRNA Analysis

We evaluated whether, in our model of early diabetes, changes occurred in specific miRNAs known to modulate the expression of genes related to calcium dynamics and SIRT1. In particular, miRNA1 overexpression in cultured cardiomyocytes silenced sodium–calcium exchange [22] and L-type calcium channel protein expression [21]. miRNA22 and miRNA34a were also selected because SIRT1, an important epigenetic regulator essential for cardiac function, was identified as a potential target. SIRT1 mRNA was repressed by miRNA22 overexpression and, even if moderately, both the SIRT1 transcript and protein increased in the hearts of miRNA22 knockout mice [19]. MiRNA34a was also found to inhibit SIRT1 expression through an miRNA34a binding site within the 3′ UTR of SIRT1 in HeLa and HEK293 cell cultures [20].

**Figure 7:**
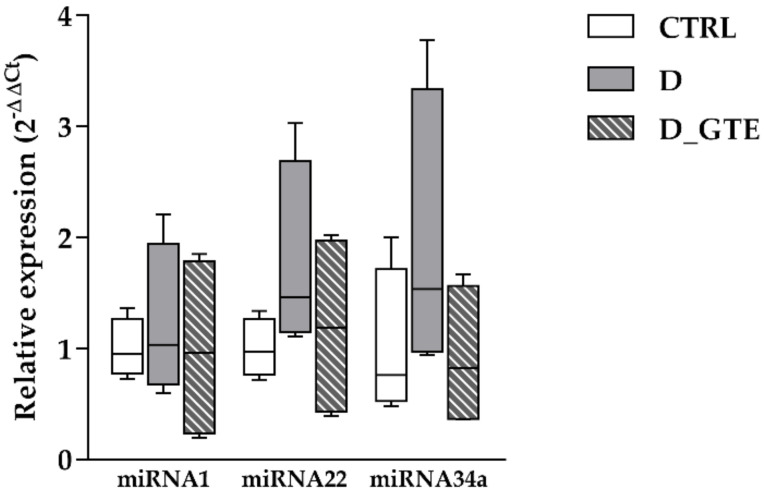
Differences in microRNA expression between the control (CTRL), diabetic (D) and GTE-treated groups (D_GTE), measured through TaqMan MicroRNA Assays for miRNA1 (rno-miR1-3p), miRNA22 (rno-miR22-3p) and miRNA34a (rno-miR34a-5p). Relative miRNA expression levels of the target genes were calculated as fold changes (2^−ΔΔCt^). Data are presented as box and whisker plots with bar graphs showing the median value. No statistically significant differences in the expression of the three miRNAs were notified among the three groups (Kruskal–Wallis test).

The expression levels of miRNA1, miRNA22 and miRNA34a were determined in comparison with the endogenous control, U6, in the myocardial samples derived from the left ventricle of control (CTRL, n = 4), diabetic (D, n = 3) and GTE-treated rats (D_GTE, n = 4) (Figure 7). Although the expression level of the three miRNAs did not show significant differences among groups, a definite trend towards an upregulation of miRNA22 and miRNA34a in D rats compared to CTRL was seen. Interestingly, GTE treatment completely restored the expression levels of both miRNA22 and miRNA34a.

## 4. Discussion

It is widely accepted that, in addition to cell oxidative stress and moderate inflammation, mitochondrial impairment and changes in the expression/activity of functional and regulatory proteins responsible for cardiomyocyte contractile efficiency participate in the occurrence of the DCM phenotype [1,2,32]. Recently, several microRNAs, a class of non-coding RNAs, have also been considered as playing a key role in various cardiovascular diseases, including DCM [33].

Despite the in-depth knowledge of the pathogenic molecular mechanisms underlying this multifactorial cardiac disease, and the prominent advances in diabetes prevention and treatment, detrimental cardiovascular complications still remain severe in diabetic subjects. Until recently, the available therapies to prevent diabetes-induced cardiac damage have had limited success and many efforts have been devoted to the development of new adjuvant cardioprotective strategies able to counteract the evolution towards diabetic cardiomyopathy [9].

With this in mind, we tested the efficacy of the prolonged in vivo administration of a standardized green tea extract (GTE) in preventing the initial functional changes occurring in the diabetic heart, given the documented effect of this compound in promoting mitochondrial function, even in the normal heart [16,25,34], and its capacity to affect gene expression by epigenetic mechanisms, through the modulation of different histone deacetylase activity [35,36]. 

In summary, our results indicate that GTE treatment induced an almost complete recovery of cardiomyocyte contractility, which was markedly impaired in the diabetic cells, by preserving mitochondrial function and energy availability, and modulating the expression of proteins involved in intracellular calcium dynamics, specifically SERCA2 and PLB. The increased SIRT1 levels in D_GTE cardiac cells, mediated by posttranscriptional processes, partially involving miRNA34a and miRNA22, associated with a high activity of this histone deacetylase, substantially contributed to the observed cardioprotective effects. At these initial stages of diabetes, no substantial changes in the expression of other genes involved in intracellular calcium fluxes were observed, i.e., of the sodium–calcium exchanger, the plasmalemma voltage-gated calcium channel or the sarcoplasmic reticulum Ca^2+^ release channels (ryanodine receptors). In line with these results, the expression levels of miRNA1 were comparable in the CTRL, D and D_GTE left ventricular samples. 

In accordance with previous studies obtained in the same model of early diabetes [11,12], a marked deterioration of contractile properties and intracellular calcium handling was observed in the untreated diabetic cardiomyocytes, as compared to the control group. The main alterations included a reduced fraction of shortening, consistent with the reduced amplitude of the calcium transient, a reduced rate of shortening, and a slow Ca^2+^ transient decay with a parallel decrease in the rate of cell relengthening. Several factors can be held responsible for these functional alterations in the D cell group. Firstly, this includes the reduced protein level of SERCA2, which is one of the most important players involved in cytosolic calcium clearing [37], responsible for both cell relengthening and the amount of calcium released for the subsequent contraction. This finding also confirmed previous data documenting a downregulation of SERCA2 expression in the diabetic heart, since the initial stages of the disease [12]. Secondly, the reduced ATP availability, taken as an index of initial mitochondrial dysfunction, can account for the decreased SERCA2 activity, prolonging the time required for cytosolic calcium removal and cell relengthening. Low ATP levels can also negatively affect sarcomere protein interaction, slowing down the rate of shortening. Finally, the diabetes-induced increase in the PLB protein level and the reduced phosphorylated form of PLB further contributed to the inhibition of SERCA2. PLB is a small regulator protein that modulates the active transport of calcium by SERCA2 into the lumen of the sarcoplasmic reticulum. Specifically, PLB acts as a reversible inhibitor of SERCA2, while this inhibition is relieved by phosphorylation [37]. PLB interacts physically with the SERCA2 pump, thereby inhibiting its ability to bind Ca^2+^ and move it into the SR. Furthermore, the changes in the SERCA2 and PLB expression levels in the D cells resulted in decreased values of the relative ratios of SERCA2/PLB and p-PLB/PLB, thus accounting for Ca^2+^ dysregulation, as already described in experimental models of diabetes and in patients with heart failure [38,39]. In vivo GTE treatment restored normal levels of both SERCA2 and PLB, as well as the energy availability and, consequently, the values of the SERCA2/PLB and p-PLB/PLB ratios, leading to an almost complete recovery of cardiomyocyte contractile properties.

The observed, marked cardioprotection could be ascribed, at least partially, to the effect of the GTE treatment which promoted SIRT1 protein expression and activity, and normalized the miRNA22 and miRNA34a levels. Our results confirm previous data showing that polyphenols, to which green tea catechins belong, are able to activate SIRT1 directly or indirectly in vitro and in vivo [40,41]. SIRT1 is a histone deacetylase involved in important biological processes such as cell survival, oxidative stress response and aging [42,43]. SIRT1 has also been reported to play a cardioprotective role during heart failure by modulating the expression of antioxidant enzymes and reducing ROS [44]. It is therefore conceivable that the GTE induced increases in SIRT1 levels and activity, while reducing myocardial oxidative damage and preserving mitochondrial function. The increased ATP content in D_GTE cardiomyocytes is in accordance with previous in vivo and in vitro studies showing that EGCG, the most abundant polyphenol of green tea, regulates: (i) mitochondrial function which, in turn, impacts cell bioenergetics (ATP production and anabolism); (ii) mitochondrial biogenesis through the activation of AMPK, a key metabolic regulator; (iii) mitochondrial redox level; and (iv) mitochondrial-related apoptosis [45]. GTE may also promote mitochondrial biogenesis, as already shown in previous studies describing the effects of catechins after kidney injury, diabetes and aging [46,47,48,49]. Mitochondrial biogenesis is regulated by multiple regulatory factors, among which PGC1-α is a master regulator. The activity of PGC1-α is modulated by AMPK and SIRT1 through phosphorylation and deacetylation, respectively. We thus hypothesized that PGC-1a activity was increased in D_GTE cardiomyocytes, as a consequence of SIRT1 increased activity, promoting mitochondrial biogenesis. This result was supported by the increase in mitochondrial mass index in D_GTE cardiomyocytes, as indicated by the GTE-induced recovery of citrate synthase activity, which was significantly reduced in the D group. This hypothesis is in accordance with previous proposed mechanisms for the SIRT1 regulation of mitochondrial biogenesis [50].

SIRT1 is also known to modulate the expression of proteins involved in intracellular calcium handling [7,12], as we actually observed in the D_GTE cardiomyocytes. Specifically, SIRT1 activation was shown to increase the expression of SERCA2 and reduce PLB levels, thus restoring the efficiency of the intracellular calcium fluxes. SIRT1 activation can also directly improve the activity of SERCA2 by protein deacetylation. As previously reported [7], acetylation of SERCA2 is significantly increased in failing human and animal hearts, and correlated with reduced SERCA2 activity. Acetylation of SERCA2 is mediated by p300, and SIRT1 is able to reverse this modification. 

Finally, it should be considered that SIRT1 is a posttranslational regulator modulating inflammation [51]; thus, it cannot be ruled out that the anti-inflammatory effect of GTE-induced SIRT1 activation played a role in the observed cardioprotection. However, in our model of early diabetes, only moderate tissue inflammation was documented, mainly characterized by the increased expression of the monocyte chemoattractant protein-1 (MCP-1) and fractalkine, in both cardiomyocytes and fibroblasts [10], while more severe tissue and systemic inflammation occur in the advanced stages of diabetic cardiomyopathy, as well as in other dysmetabolic pathologies [52].

## 5. Conclusions

In conclusion, our results demonstrate that, in this rat model of initial diabetes, the prolonged in vivo administration of a standardized GTE increased the expression and activity of SIRT1, leading to a recovery of mitochondrial function and intracellular ATP availability, and restored the normal expression levels of proteins involved in intracellular calcium dynamics. Collectively, these beneficial effects prevented cardiomyocyte contractile impairment.

These findings support the idea that dietary green tea polyphenols can constitute a promising adjuvant strategy for counteracting the initial myocardial damage occurring in the diabetic heart and its progression towards overt DCM. Our data also indicate that SIRT1 may serve as a potential therapeutic target for the management of DCM.

## Figures and Tables

**Table 1:** Specific primers used for qPCR amplification.

	Forward Primer Sequence	Reverse Primer Sequence
**SERCA2**	5′ AACTACCTGGAGCCTGCAAT 3′	5′ TTCCCCAAGCTCAGTCATGC 3′
**PLN**	5′ CATGCCAACGCAGTTACAACCT 3′	5′ TCGTGACCCTTCACGACGAT 3′
**RYR2**	5′ GGCGGAATTTCTTGCCAAC 3′	5′ CCTCGCACCTCATCCTGAGT 3′
**NCX1**	5′ CGAAATGGATGGGAAAGTAGTCAAC 3′	5′ TCTTTGTCGGGATGCTTCTGC 3′
**CACNA1C**	5′ ATGGTTCTTGTCAGCATGTTGCGG 3′	5′ TGCAAATGTGGAACCGGTGAAGTG 3′
**SIRT1**	5′ GTCTGTGCCTTCCAGTTGCT 3′	5′ CTGCTTGCTGTCCATACCTG 3′
**GAPDH**	5′ GTTCCAGAGACAGCCGCATC 3′	5′ CGTTCACACCGACCTTCACC 3′

**Table 2:** Mean values ± SEM of blood glucose levels (mg/dL) measured in the CTRL group, and untreated (D) and GTE-treated (D_GTE) diabetic animals, at different time points during the experimental protocol. ** *p* < 0.01 vs. CTRL (Two-way ANOVA).

	CTRL	D	D_GTE
**Day 0**	103 ± 2	107 ± 6	105 ± 3
**Day 2**	105 ± 4	356 ± 11 **	395 ± 28 **
**Day 14**	101 ± 5	424 ± 25 **	448 ± 19 **
**Day 28**	97 ± 5	472 ± 32 **	487 ± 3 **

**Table 3:** Mean values ± SEM of body weights (g) measured weekly in the CTRL group, and untreated (D) and GTE-treated (D_GTE) diabetic animals, at different time points during the experimental protocol. ** *p* < 0.01 vs. CTRL (Two-way ANOVA).

	CTRL	D	D_GTE
**Day 0**	359 ± 9	378 ± 5	384 ± 7
**Day 2**	370 ± 9	355 ± 5	364 ± 8
**Day 14**	382 ± 13	332 ± 6 **	335 ± 7 **
**Day 28**	387 ± 14	331 ± 8 **	336 ± 9 **

## Data Availability

Data is contained within the article.
